# The impact of main *Areca Catechu* root exudates on soil microbial community structure and function in coffee plantation soils

**DOI:** 10.3389/fmicb.2023.1257164

**Published:** 2023-10-20

**Authors:** Shaoguan Zhao, Ang Zhang, Qingyun Zhao, Yunping Dong, Lanxi Su, Yan Sun, Feifei Zhu, Dangling Hua, Wu Xiong

**Affiliations:** ^1^Spice and Beverage Research Institute, Chinese Academy of Tropical Agricultural Science, Wanning, China; ^2^College of Agricultural Resources and Environmental Sciences, Henan Agricultural University, Zhengzhou, China; ^3^Sanya Research Institute, Chinese Academy of Tropical Agricultural Science, Sanya, China; ^4^Jiangsu Provincial Key Lab of Solid Organic Waste Utilization, Jiangsu Collaborative Innovation Center of Solid Organic Wastes, Educational Ministry Engineering Center of Resource-Saving Fertilizers, Nanjing Agricultural University, Nanjing, China

**Keywords:** continuous cropping obstacle, soil physic-chemical properties, soil enzyme activity, microbial community structure, functional prediction

## Abstract

Coffee is an important cash crop worldwide, but it has been plagued by serious continuous planting obstacles. Intercropping with *Areca catechu* could alleviate the continuous planting obstacle of coffee due to the diverse root secretions of *Areca catechu*. However, the mechanism of *Areca catechu* root secretion in alleviating coffee continuous planting obstacle is still unclear. The changes of coffee rhizosphere soil microbial compositions and functions were explored by adding simulated root secretions of *Areca catechu*, the primary intercropping plant species (i.e., amino acids, plant hormone, organic acids, phenolic acids, flavonoids and sugars) in current study. The results showed that the addition of coffee root exudates altered soil physicochemical properties, with significantly increasing the availability of potassium and organic matter contents as well as promoting soil enzyme activity. However, the addition of plant hormone, organic acids, or phenolic acids led to a decrease in the Shannon index of bacterial communities in continuously planted coffee rhizosphere soil (RS-CP). The inclusion of phenolic acids specifically caused the decrease of fungal Shannon index. Plant hormone, flavonoids, phenolic acids, and sugars increased the relative abundance of beneficial bacteria with reduced bacterial pathogens. Flavonoids and organic acids increased the relative abundance of potential fungal pathogen *Fusarium*. The polyphenol oxidase, dehydrogenase, urease, catalase, and pH were highly linked with bacterial community structure. Moreover, catalase, pH, and soil-available potassium were the main determinants of fungal communities. In conclusion, this study highlight that the addition of plant hormone, phenolic acids, and sugars could enhance enzyme activity, and promote synergistic interactions among microorganisms by enhancing the physicochemical properties of RS-CP, maintaining the soil functions in coffee continuous planting soil, which contribute to alleviate the obstacles associated with continuous coffee cultivation.

## 1. Introduction

As a major cash crop worldwide, coffee plays a crucial role in international agricultural trade (Higashi, 2019). However, the sustainability of coffee production is at risk due to continuous cropping obstacles (CCO) which can cause a decline in coffee quality and production over time (Xiong et al., 2015; Zhao et al., 2018; Su et al., 2022). The CCO phenomenon is closely associated with deteriorating soil properties, the accumulation of allelopathic substances, and microbial imbalances (Xiong et al., 2018; Zhao et al., 2021; Li Q. et al., 2022). It is therefore imperative to investigate the interactions among root secretion, soil properties, and microbiome to maintaining the health of coffee rhizosphere soil of coffee rhizosphere soil and mitigate the negative effects of CCO on coffee production.

Continuous cultivation of coffee can decrease soil organic carbon content and porosity, proliferation of pests and diseases in the topsoil of coffee plantations, and reduced plant adaptability (Ajayi et al., 2021; Rezende et al., 2021; Ayalew et al., 2022). Under long-term continuous cultivation, the root exudates of coffee, such as phenolic compounds, can potentially have toxic effects on certain microorganisms, resulting in the changes of microbial community structure (Jurburg et al., 2020). Previous research has highlighted the significance of soil physic-chemical properties remediation and soil microbiome structure regulation as key factors in alleviating CCO (Xiong et al., 2015; Cheng et al., 2020; Zeng et al., 2020; Liu Q. et al., 2022; Wang et al., 2022). Root secretions also appear to play a critical role in regulating CCO, with intercropping crop groups increasing the diversity of root exudates and optimizing soil nutrients in intercropping ecosystems, thereby promoting positive effects on soil microbial diversity and stability (Zeng et al., 2020). Intercropping systems can alleviate CCO of crops through the improvement of soil bacteria metabolism, soil enzyme activity, and the alleviation of autotoxicity via root metabolite interactions (Cheng et al., 2020; Liu Q. et al., 2022). Consequently, the intercropping system is deemed to offer stable services for crop growth by regulating the soil microbiome and reducing allelochemicals (Guo et al., 2020). *Areca catechu* is a tropical cash crop that is extensively intercropping with coffee to increase the productivity per unit area in several coffee plantations (Sujatha et al., 2011). The research team were observed that coffee plants intercropped with *Areca catechu* exhibited more vigorous growth compared to those grown solely in previous study. However, further studies need to be conducted to investigate the mechanism of intercropping with *Areca catechu* as a means of alleviating coffee CCO.

It is well established that plants play a crucial role in regulating the biological and abiotic conditions of their rhizosphere soil through root secretions, which in turn can affect plant growth by modifying rhizosphere environment (Hu et al., 2018; Zhao et al., 2021; Li M. et al., 2022). Research has revealed that root secretions can act as selective agents that potentially influence the composition of the rhizosphere microbiome (Zhalnina et al., 2018; Zhou et al., 2020). Root exudates aid in nutrient acquisition by modulating the rhizosphere microbiome and facilitating plant–soil feedback to help plants fend off pathogens (Hu et al., 2018). Root exudates mediate the proliferation of specific bacterial communities primarily by targeting plant genetics and physiology to modify the composition of soil microbial communities (Fu et al., 2021; Kawasaki et al., 2021). For example, phloridzin could attract specific pathogenic microorganisms that cause sequential crop diseases (Hofmann et al., 2009); the addition of various organic acids leads to a reduction in soil microbial community richness and diversity index, ultimately leading to specific changes in microbial community structure (Ma et al., 2019); Phenolic acids can decrease soil ecosystem stability by reducing beneficial microbial communities and increasing harmful microbial communities (Huang et al., 2019). Plants are capable of adjusting the composition of their secretions at different stages of growth (Upadhyay et al., 2022). Despite the existence of various types of secretions, the effects of different secretions on soil physicochemical properties and microbial community composition remain unclear.

In this study, we conducted a field-controlled experiment to investigate the effects of primary root exudates from *Areca catechu* on the physicochemical properties, enzyme activity, microbial community, and their interactions in the rhizosphere soil of coffee plants. We aim to answer the following scientific questions: (1) Investigate which type of *Areca catechu* root exudate has a greater impact on the coffee rhizosphere microbiota; (2) Reveale the main driving factors of *Areca catechu* root exudate regulating coffee rhizosphere microorganisms.

## 2. Materials and methods

### 2.1. Experiment site

The experiment was conducted in the planting resource garden of the Spice Beverage Research Institute located in Hainan Province, China (110°6′ E, 18°24′ N). The soil in the experimental site showed a soil organic carbon (SOM) content of 164.2 g/kg, an alkali-hydrolyzed nitrogen content (AN) of 98.5 g/kg, a soil-available phosphorus content (AP) of 250.2 mg/kg, and a soil-available potassium content (AHK) of 84.1 mg/kg.

### 2.2. Experimental material

The soil was collected from a coffee plantation with a long history of monocropping coffee. The topsoil was excavated, and the coffee roots were dug out. The roots were gently shaken to remove non-rhizosphere soil, and then the roots with rhizosphere soil were placed in a bag and vigorously shaken to obtain the rhizosphere soil of the coffee. A sieve (<2 mm) was used to remove plant debris and fully homogenize it for the experiment.

According to previous studies, root exudates of *Areca catechu* were analyzed and determined by using liquid chromatography-mass spectrometry technology. From 6 different categories, 20 compounds with relatively high content were selected based on accessibility and relative abundance to represent the main root exudate composition of *Areca catechu* for subsequent experiments.

### 2.3. Experimental design

Eight treatments were added with different types of root secretions: sterile water (CK), 20% sterile methanol solution (KCK), 20% sterile methanol solution dissolved organic acids (OA), 20% sterile methanol solution dissolved amino acids (AMA), 20% sterile methanol solution dissolved phenolic acids (PA), 20% sterile methanol solution dissolved flavonoids (FLA), 20% sterile methanol solution dissolves sugars (SUG), 20% sterile methanol solution dissolves plant hormone (AUX). Each treatment was repeated six times (Supplementary Table S1).

A randomized block group design was used in the experiment. Each treatment was replicated six times, with each replicate consisting of 600 g of soil placed in large containers. The packaged soil was placed in coffee fields to ensure uniform environmental conditions. Before each addition, the prepared exogenous secretion solutions are adjusted to neutral pH using sodium hydroxide to eliminate the strong interference of soil pH on microbial communities. Subsequently, the solutions were added to the packaged soil in the coffee fields. The daily input of carbon from other crops in sandy soil is used to estimate the amount of root exudates secreted by *Areca catechus*: 0.05–0.1 mg C/g per day (Trofymow et al., 1987; Iijima et al., 2000; Baudoin et al., 2003), as few studies have reported the daily carbon input from *Areca catechu* tree roots into the soil. The exogenous secretion added per unit soil was 0.075 mg C/g/d. In each simulated root exudate, the carbon content of each carbon source material in the solution was equal (e.g., the carbon content of citric acid, succinic acid, and tartaric acid accounted for 1/3 of the total carbon content in each added solution, excluding the carbon content in methanol). After 6 weeks of cultivation, destructive sampling was conducted, and the soil samples were stored at −80°C.

### 2.4. Analysis of soil physic-chemical properties and soil enzyme activities

Soil pH was measured with a pH meter (FE28, China; soil and water ratio 1:2.5). soil-available phosphorus (AP), soil-available potassium (AK), alkali-hydrolyzed nitrogen (AHN), and soil organic matter (SOM) were separately measured by the molybdenum blue, flame photometry, potassium persulfate oxidation, and dichromate oxidation method, respectively (Lu, 1999). The concentrations of soil urease (S-UE), soil dehydrogenase (S-DHA), soil cellulase (S-CL), soil catalase (S-CAT), peroxidase (S-POD), soil polyphenol oxidase (S-PPO), soil acid phosphatase (S-ACP) and soil alkaline phosphatase (S-ALP) were determined by micro method kits from Suzhou Grace Biotechnology Co., Ltd. with the use of an enzyme marker (SynergyH1, USA).

### 2.5. Soil DNA extraction and sequencing

Soil microbial DNA was extracted three times from 0.5 g fresh soil by using the EZNA® Soil DNA Extraction Kit (Omega, USA). The 0.8% agarose gels was used to check the purity and quality of genomic DNA. The DNA was extracted using sequences: 338F (5′-ACTCCT ACGGGAGGCAGCA-3′) and 806R (5′-GGACTACHVGGGT WTCTAAT-3′) bacterial primers with barcode tags (Song et al., 2020). Fungal internal transcribed spacer (ITS1) genes were amplified using the ITS5F (5′-GGAAGTAAAAGTCGTAACAAGG-3′) and ITS1R (5′- GCTGCGTTCTTCATCGATGC-3′) barcode primers, with the amplification program according to the reference description (Zhou et al., 2021). The PCR amplification products were quantified by fluorescence with the Quant-iT PicoGreen dsDNA Assay Kit and a Microplate reader (BioTek, FLx800) and were used for quantification. Each sample was mixed in the corresponding ratio based on the sequencing volume requirement of each sample, based on the results of the fluorescence quantification. Sequencing libraries were prepared using the Illumina TruSeq Nano DNA LT Library Prep Kit, which was used for sequencing, followed by double end sequencing (Paired-end) of DNA fragments from the community using the Illumina platform.

### 2.6. Bioinformatics analysis

Microbiome bioinformatics were performed with QIIME 22019.4 (Bolyen et al., 2019) with slight modification according to the official tutorials.^1^ Briefly, raw sequence data were demultiplexed using the demux plugin following by primers cutting with cutadapt plugin (Martin, 2011). Sequences were then quality filtered, denoised, merged and chimera removed using the DADA2 plugin (Callahan et al., 2016). Non-singleton amplicon sequence variants (OTUs) were aligned with maff and used to construct a phylogeny with fasttree2 (Price et al., 2010). Taxonomy was assigned to OTUs using the classify-sklearn naïve Bayes taxonomy classifier in feature-classifier plugin (Bokulich et al., 2018) against the Greengenes 13_8 99% OTUs reference sequences (Mcdonald et al., 2012). Alpha-diversity metrics Shannon, beta diversity metrics weighted UniFrac (Lozupone et al., 2007) unweighted UniFrac (Lozupone and Knight, 2005), Jaccard distance, and Bray–Curtis dissimilarity were estimated using the diversity plugin with samples were rarefied to sequences per sample.

### 2.7. Statistical analysis

All statistical analyses were performed in R 4.2.0 and visualized with ggplot2. Principal coordinate analysis (PCoA) based on Bray-Curtis distance was used to explore differences of several root secretions on bacterial and fungal community structure, respectively. Soil physicochemical properties, soil enzyme activity, soil microbial diversity indices, relative abundance of specific microbial taxa, and relative abundance of soil microbial functional groups were analyzed using one-way ANOVA to determine differences among the different root secretions addition. The statistical significance (*p* < 0.05) was calculated using Tukey test. Correlations between soil properties, soil enzyme activity and soil microbial community diversity were calculated and analyzed using Spearman correlation matrices. FAPROTAX (Liang et al., 2020) and FUNGuild (Jiang et al., 2021) are commonly used to predict the functions of bacterial and fungal communities.

## 3. Results

### 3.1. Changes of soil physical and chemical properties and enzyme activities

One-way ANOVA analysis revealed significant effects of exogenous secretion addition on soil properties and enzyme activities. There were significant changes in soil-available potassium and phosphorus between the CK treatment and the KCK treatment. Soil-available potassium showed a significant increase of 22.61%, while soil-available phosphorus exhibited a significant decrease of 35.24%. Compared to the control treatment (KCK), the addition of AMA significantly increased soil organic matter, alkali-hydrolyzed nitrogen, and soil-available potassium by 66.44, 241.73, and 11.52%, respectively. The soil-available potassium and alkali-hydrolyzed nitrogen levels were significantly increased by 27.06 and 37.65%, respectively, under the AUX addition treatment. Treatment with FLA significantly increased organic matter by 23.79%. The addition of OA significantly increased organic matter and pH by 29.48 and 69.93%, respectively. In the PA treatment, pH and alkali-hydrolyzed nitrogen were significantly higher by 47.06 and 18.32%, respectively. The addition of SUG significantly increased organic matter, pH, and alkali-hydrolyzed nitrogen levels. A significant increase of 38.00, 42.48, and 17.85% was observed in organic matter, pH, and soil-available potassium of the SUG treatment. However, the soil-available phosphorus content was significantly reduced by 49.20 and 36.54% in the AMA and PA, treatments, respectively (all *p* < 0.05, Table 1).

**Table 1:** Effects of different root secretions on soil physic-chemical properties.

Treatments	SOM (mg/kg)	pH	AK (mg/kg)	AP (mg/kg)	AHN (mg/kg)
CK	169.17 ± 8.62^e^	4.68 ± 0.02^f^	75.42 ± 0.75^f^	30.76 ± 35.34^a^	91.93 ± 1.44^e^
KCK	186.6 ± 9.7^de^	4.59 ± 0.07^f^	92.47 ± 1.3^e^	19.92 ± 39.9b^c^	91.7 ± 1.71^e^
AMA	310.57 ± 31.35^a^	5.03 ± 0.2^e^	103.12 ± 0.75^c^	10.12 ± 25.98^d^	313.37 ± 10.61^a^
AUX	200.27 ± 0.42^d^	5.39 ± 0.07^d^	117.5 ± 1.99^a^	22.09 ± 18.97^b^	126.23 ± 1.75^b^
FLA	231 ± 1.93^c^	5.57 ± 0.04^d^	97.79 ± 5.27^d^	17.96 ± 38.06^c^	100.57 ± 0.87^d^
OA	241.6 ± 12.23^bc^	7.8 ± 0.14^a^	99.93 ± 1.99^cd^	18.73 ± 19.73^bc^	96.83 ± 3.15^de^
PA	201.8 ± 1.3^d^	6.75 ± 0.03^b^	101.52 ± 4.19^c^	12.64 ± 3.57^d^	108.5 ± 1.71^c^
SUG	257.5 ± 20.36^b^	6.54 ± 0.36^c^	108.98 ± 0.75^b^	16.42 ± 15.47^c^	100.57 ± 1.75^d^

SOM (mg/kg), soil organic matter; AK (mg/kg), soil-available potassium; AP (mg/kg), soil-available phosphorus; AHN (mg/kg), alkali-hydrolyzed nitrogen. Different letters indicate significant differences between treatments (p < 0.05). *n* = 6. CK, control (sterile water); KCK, 20% sterile methanol solution; AMA, amino acids; AUX, auxin; FLA, flavonoids; OA, organic acids; PA, phenolic acids; SUG, sugar. Different letters indicate significant differences between treatments under the same soil physicochemical properties, *n* = 6.

There were significant differences in the activities of cellulase, urease, hydrogen peroxide enzyme, peroxidase, and acid phosphatase between the CK treatment and the KCK treatment. The activity of urease, hydrogen peroxide enzyme, and peroxidase in the KCK treatment showed a significant increase of 6.90, 9.08, and 26.39% respectively, while the activities of cellulase and acid phosphatase exhibited a significant decrease of 79.10 and 81.50%, respectively. Compared to the KCK treatment, the activities of polyphenol oxidase, dehydrogenase, and peroxidase were significantly increased by 83.90, 90.35, and 83.17%, respectively, under the AMA addition treatment. The addition of OA increased the activities of soil polyphenol oxidase, peroxidase, and dehydrogenase by 144.07, 56.12, and 97.26%, respectively. In the PA treatment, catalase, peroxidase, and dehydrogenase activities were significantly increased by 37.72, 54.66, and 61.13%, respectively. The SUG addition significantly increased polyphenol oxidase and peroxidase activities by 105.08 and 111.45%, respectively (all *p* < 0.05, Table 2).

**Table 2:** Effects of different root secretions on soil enzyme activities.

Treatments	S-PPO nmol/h	S-CL μg/d/g	S-UE μg/d/g	S-CAT μmol/h/g	S-POD nmol/h/g	S-DHA μg/d/g	S-ACP nmol/h/g	S-ALP nmol/h/g
CK	212.6 ± 9.19^c^	210.82 ± 47.72^c^	646.97 ± 40.33^c^	273 ± 13.03^e^	1808.87 ± 92.82^e^	1215.98 ± 79.91^e^	1514.22 ± 205.09^b^	811.56 ± 58.98^bcd^
KCK	241.42 ± 6.74^c^	117.74 ± 16.62^d^	695.18 ± 7.01^b^	300.25 ± 3.82^d^	2457.47 ± 6.74^cd^	1212.04 ± 171.92^e^	834.28 ± 341.68^c^	561.21 ± 100.76^d^
AMA	390.96 ± 95.1^b^	1116.3 ± 90.84^a^	357.36 ± 31.43^e^	351.06 ± 21.23^c^	3313.26 ± 500.15^b^	2314.66 ± 285.28^a^	3079.89 ± 484.61^a^	513.63 ± 93.89^d^
AUX	221.6 ± 49.14^c^	184.43 ± 31.1^c^	432.89 ± 5.32^d^	264.82 ± 28.78^e^	1830.49 ± 120.89^e^	1613.04 ± 16.48^c^	1429.23 ± 61.01^b^	903.61 ± 36.03^bc^
FLA	228.81 ± 40.04^c^	162.44 ± 49.44^cd^	670.43 ± 19.32^bc^	314.85 ± 27.24^d^	2171.01 ± 49.21^de^	1299.46 ± 317.56^de^	1451.6 ± 178.15^b^	1525.7 ± 143.95^a^
OA	518.88 ± 50.9^a^	623.8 ± 28.9^b^	775.77 ± 22.63^a^	426.2 ± 0.83^a^	2599.8 ± 631.31^cd^	2398.69 ± 210.21^a^	1245.82 ± 329.69^bc^	1397.63 ± 652.66^a^
PA	279.26 ± 20.86^c^	174.54 ± 9.08^cd^	636.85 ± 2.19^c^	375.98 ± 6.2^b^	2797.63 ± 61.32^c^	1959.34 ± 20.86^b^	1581.32 ± 279.43^b^	586.12 ± 127.54^cd^
SUG	436 ± 67.84^b^	155.48 ± 23.62^cd^	703.18 ± 45.72^b^	347.95 ± 1.26^c^	3824.94 ± 475.85^a^	1481.06 ± 4.85^cd^	1321.87 ± 516.63^b^	1081.03 ± 33.65^b^

S-PPO (nmol/h/g), soil polyphenol oxidase; S-CL (μg/d/g), soil-cellulase; S-UE (μg/d/g), soil urease; S-CAT (μmol/h/g), soil catalase; S-POD (nmol/h/g), soil peroxidase; S-DHA (μg/d/g), soil dehydrogenase; S-ACP (nmol/h/g), soil acid phosphatase; S-ALP (nmol/h/g), soil alkaline phosphatase. CK, control (sterile water), KCK, 20% sterile methanol solution; AMA, amino acids; AUX, auxin; FLA, flavonoids; OA, organic acids; PA, phenolic acids; SUG, sugar. Different letters indicate significant differences between treatments under the same soil enzyme activity, *n* = 6, treatments abbreviations and indicator abbreviations see Table 1.

### 3.2. Changes in diversity indices of bacterial and fungal communities upon addition of *Areca catechu* main root secretions

**Figure 1:**
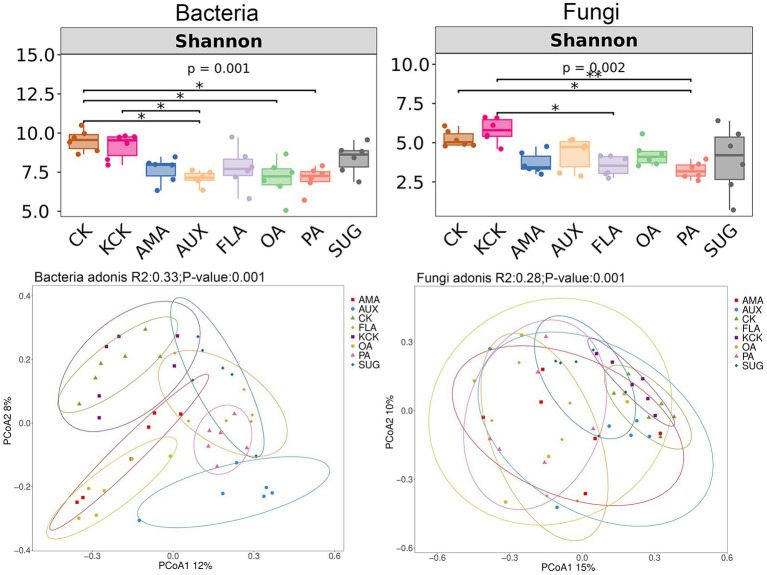
Diversity of bacterial and fungal communities in rhizosphere soil. *n* = 6. CK, control (sterile water); KCK, 20% sterile methanol solution; AMA, amino acids; AUX, auxin; FLA, flavonoids; OA, organic acids; PA, phenolic acids; SUG, sugar. * Correlation is significant at the 0.05 level; ** Correlation is significant at the 0.01 level; and *** Correlation is significant at the 0.001 level.

The one-way ANOVA analysis revealed significant effects of different root secretion additions on soil microbial Shannon index. There were no significant differences in Shannon index of soil bacteria and fungi communities between the CK and KCK treatments. In the bacterial community, compared to CK, the Shannon index was significantly reduced by 25.18, 26.44, and 25.50% in the AUX, OA, and PA treatments, respectively. Additionally, compared to KCK, the AUX treatment resulted in a significant reduction of 22.20% in Shannon index. In the fungal community, the PA treatment exhibited a significant reduction of 38.38% in Shannon index compared to CK, and the FLA and PA treatments exhibited significant reductions of 15.03 and 22.54%, respectively, compared to KCK (all *p* < 0.05, Figure 1).

The bacterial community Shannon index significantly and negatively correlated with S-PPO (*p* < 0.05), S-DHA (*p* < 0.01), pH (*p* < 0.01), and AHN (*p* < 0.01). Similarly, the fungal community Shannon index was significantly and negatively correlated with S-CAT (*p* < 0.05), S-DHA (*p* < 0.01), pH (*p* < 0.01), SOM (*p* < 0.05), and AHN (*p* < 0.01). Soil microbial diversity indices were also significantly and negatively correlated with soil AP (*p* < 0.05) (Supplementary Table S3).

Principal Coordinate Analysis (PCoA) was used to exploring the dissimilarity in community composition between treatments. There were no significant differences in bacterial and fungal community structure between the CK and KCK treatments. The addition of FLA, AMA, OA, AUX, and PA treatments had a significant effect on the bacterial community structure when compared to KCK. There was no significant difference in the bacterial community structure between the SUG addition treatment and KCK treatment. Similarly, the fungal community structures under the PA and FLA addition treatments were significantly different from KCK, while there was no significant difference in the fungal community structures between the AMA, AUX, OA, SUG, and KCK treatments (all *p* < 0.05, Figure 1).

### 3.3. Effect of *Areca catechu* main root secretions addition on the major bacterial communities

**Figure 2:**
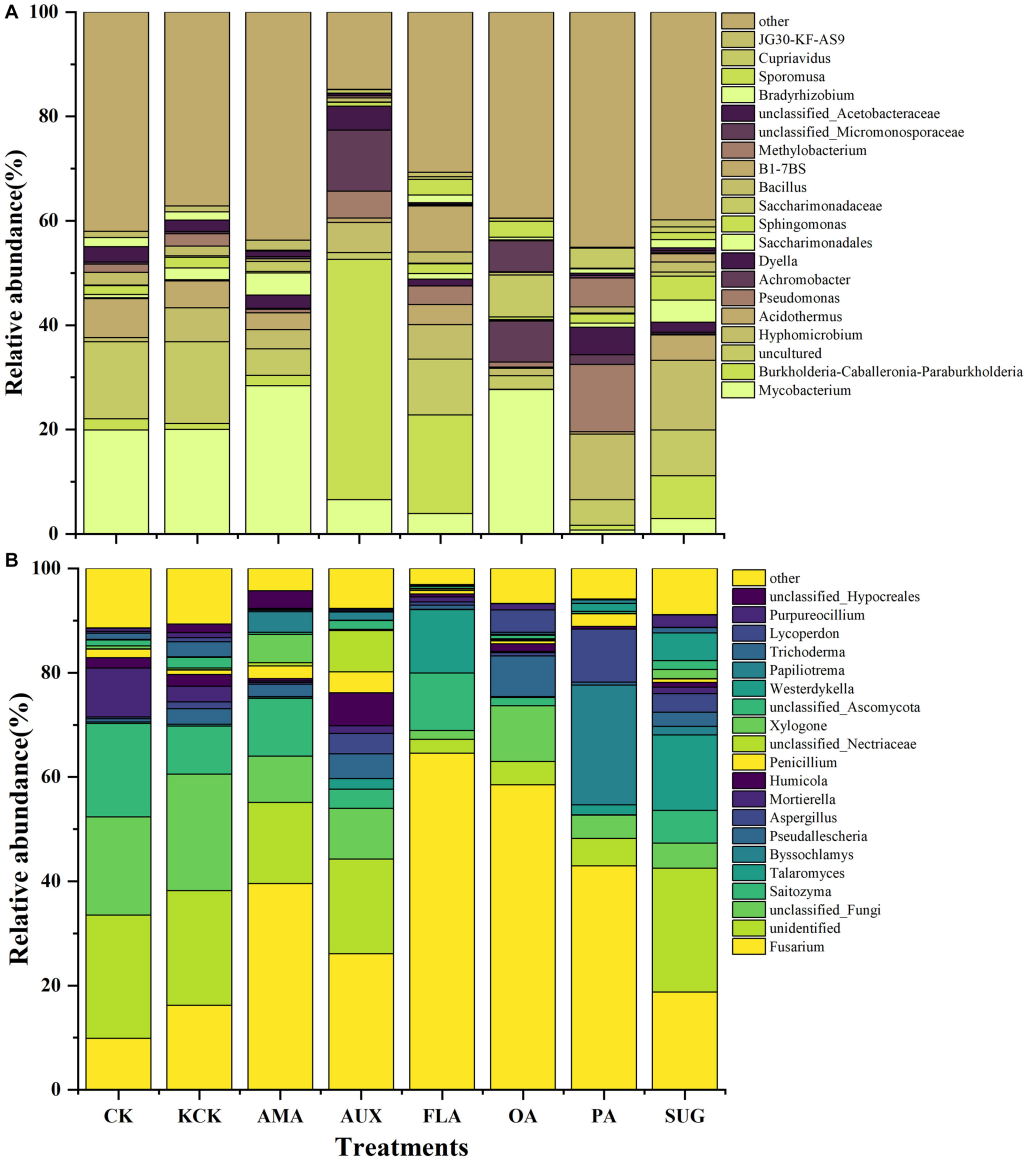
Relative abundance of top 20 genera of bacterial **(A)** and fungal **(B)** microbial communities in rhizosphere soil. *n* = 6. CK, control (sterile water); KCK, 20% sterile methanol solution; AMA, amino acids; AUX, auxin; FLA, flavonoids; OA, organic acids; PA, phenolic acids; SUG, sugar.

In the CK and KCK treatments, the major bacterial communities. (relative abundance >1%) were *Mycobacterium*, *Burkholderia*-*Caballeronia*-*Paraburkholderia*, *Acidothermus*, *Sphingomonas*, *Bacillus*, *Methylobacterium*, *Bradyrhizobium*, and *JG30*-*KF*-*AS9*, and there was no significant difference in the relative abundance of these bacterial genera between the two treatments. Compared to KCK, the addition of AMA significantly reduced *Bacillus* and *Bradyrhizobium* by 72.47 and 97.00%, respectively (*p* < 0.001). The addition of AUX increased *Burkholderia*-*Caballeronia*-*Paraburkholderia* by 3866.61% (*p* < 0.001), while significantly reducing *Mycobacterium*, *Acidothermus*, *Bacillus*, and *Bradyrhizobium* by 67.22% (*p* < 0.01), 83.54% (*p* < 0.01), 56.24% (*p* < 0.01), and 90.66% (*p* < 0.001), respectively. In the FLA treatment, *Burkholderia*-*Caballeronia*-*Paraburkholderia* was significantly increased by 1513.02% (*p* < 0.001), while *Mycobacterium* was significantly decreased by 80.80% (*p* < 0.01). The addition of OA significantly reduced *Acidothermus*, *Bacillus*, *Bradyrhizobium*, and *JG30*-*KF*-*AS9* by 95.59% (*p* < 0.01), 74.91% (*p* < 0.01), 67.98% (*p* < 0.05), and 92.83% (*p* < 0.05), respectively. In the PA treatment, *Methylobacterium* was significantly increased by 131.51% (*p* < 0.001), while *Mycobacterium*, *Acidothermus*, and *Bacillus* were significantly reduced by 96.51% (*p* < 0.01), 90.91% (*p* < 0.01), and 34.68% (*p* < 0.05), respectively. The addition of SUG significantly decreased *Mycobacterium* by 85.56% (*p* < 0.01) while increasing *Sphingomonas* by 126.12% (*p* < 0.001). *Hyphomicrobium* became the dominant genus in all treatments, except the KCK treatment, and significantly increased by 91.99% (*p* < 0.001) and 104.24% (*p* < 0.001) in the PA and SUG treatments, respectively (Figure 2; Supplementary Table S2).

For the fungal community, the dominant genera were *Fusarium*, *Saitozyma*, *Mortierella*, *Trichoderma*, and *Penicillium* in CK and KCK treatments, and there was no significant difference in the relative abundance of these bacterial genera between the two treatments. Compared with KCK, the *Mortierella* treated by AMA was significantly reduced by 92.29% (*p* < 0.001); *Saitozym* and *Mortierella* were significantly reduced by 60.18% (*p* < 0.05) and 49.34% (*p* < 0.01) as a result of AUX treatment; *Fusarium* under FLA treatment was significantly increased by 298.75% (*p* < 0.001), *Trichoderma* and *Mortierella* were significantly decreased by 97.00% (*p* < 0.05) and 75.73% (*p* < 0.01); *Fusarium* treated with OA was significantly increased by 261.24% (*p* < 0.001), *Saitozyma* and *Mortierella* were significantly decreased by 83.06 and 99.40% (*p* < 0.001); *Saitozyma*, *Mortierella* was significantly reduced by 99.49 and 99.81% (*p* < 0.001) in the PA treatment group; *Mortierella* significantly reduced by 57.86% (*p* < 0.001) in treatment SUG (Figure 2; Supplementary Table S2).

Compared to the control group (KCK), the addition of *Areca catechu* main root secretions in coffee rhizosphere soil demonstrated inhibitory effects on potential pathogenic genera, such as *Mycobacterium* and *Acidothermus*, within the bacterial community. Furthermore, the inclusion of AUX, FLA, PA, and SUG led to a significant increase in the relative abundance of two potential beneficial genera, namely *Burkholderia*-*Caballeronia*-*Paraburkholderia* and *Sphingomonas*. Notably, the PA and SUG treatments resulted in the emergence of a new dominant potential beneficial genus, *Hyphomicrobium*. Within the fungal community, the FLA and OA treatments significantly elevated the relative abundance of the potential pathogenic genus *Fusarium*. Additionally, the overall relative abundance of the potential beneficial genus *Mortierella* experienced a significant reduction across all treatments (Supplementary Tables S2, S3).

### 3.4. Correlation of soil physicochemical properties and enzyme activities with microbial communities

**Figure 3:**
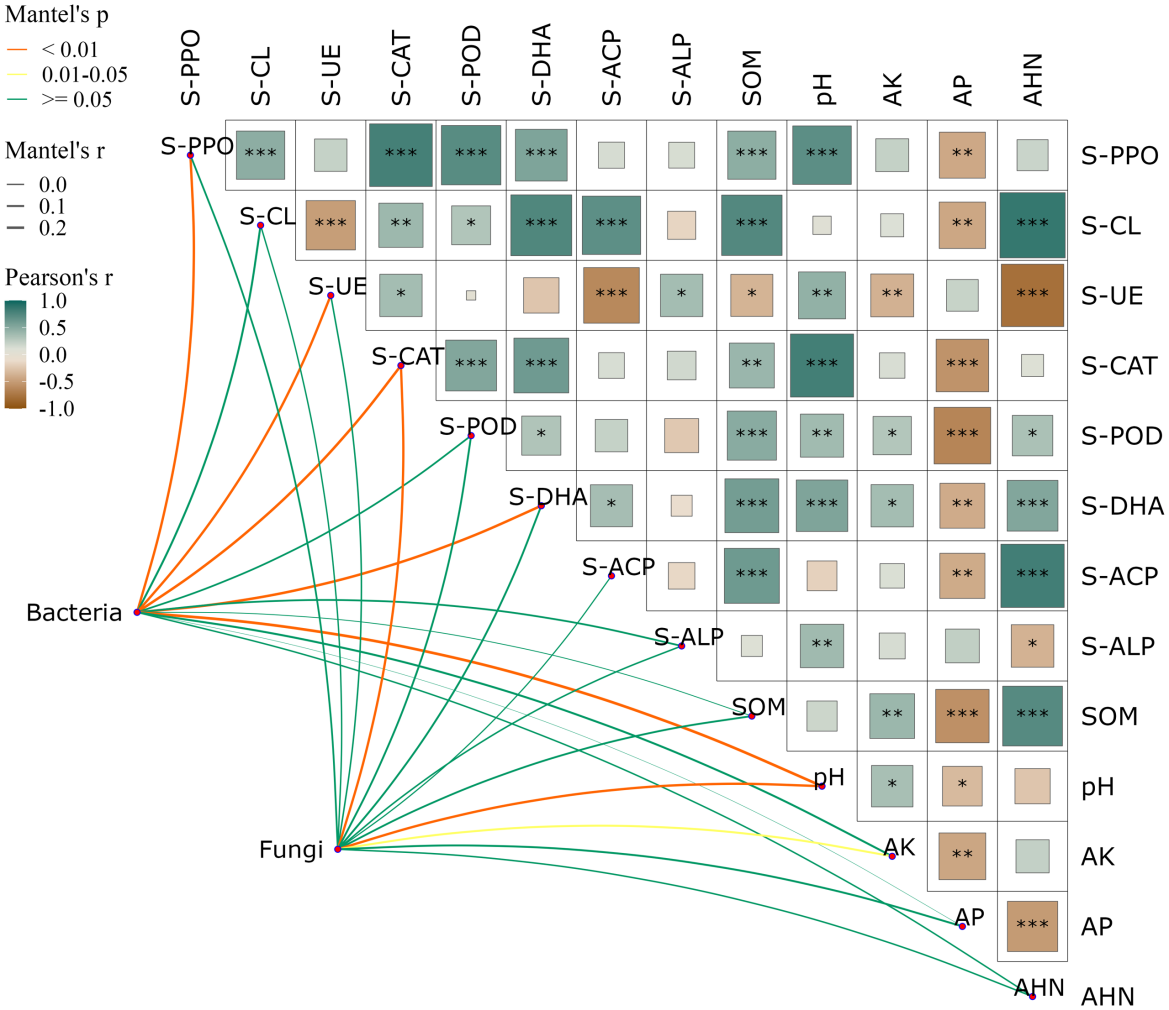
Effects of soil physic-chemical and enzyme activities on relative abundance of bacterial **(A)** and fungi **(B)** communities at the genus level. Heatmap showing the correlation between soil properties, network diagram shows the correlation between soil microbial community and soil properties. Red represents positive correlation, blue represents negative correlation, the darker the color, the stronger the correlation. The symbol “*” indicates statistical significance at *p* < 0.05, “**” indicates *p* < 0.01, and “***” indicates *p* < 0.001.

Significant positive correlations were found between soil SOM and S-PPO (*R* = 0.60, *p* < 0.001), S-CAT (*R* = 0.56, *p* < 0.01), S-POD (*R* = 0.56, *p* < 0.001) and S-DHA (*R* = 0.61, *p* < 0.001), and significant negative correlations were found with S-UE (*R* = 0.01, *p* < 0.05). pH and S-PPO (*R* = 0.64, *p* < 0.001), S-CAT (*R* = 0.72, *p* < 0.001), S-POD (*R* = 0.39, *p* < 0.01) and S-DHA (*R* = 0.53, *p* < 0.001). AP exhibited significant negative correlations with several indicators including S-PPO (*R* = −0.55, *p* < 0.01), S-CL (*R* = −0.23, *p* < 0.01), S-CAT (*R* = −0.62, *p* < 0.001), S-POD (*R* = −0.73, *p* < 0.001), S-DHA (*R* = −0.45, *p* < 0.01), and S-ACP (*R* = −0.28, *p* < 0.01). The inter-root soil bacterial community genus level structure had a significant response to soil S-PPO, S-UE, S-CAT, S-DHA, and pH, and the fungal genus level structure had a significant response to soil S-CAT, pH, and AK (Figure 3). This study demonstrates that there is a close correlation between soil physicochemical factors as well as between physicochemical factors and soil microbial community structure.

**Figure 4:**
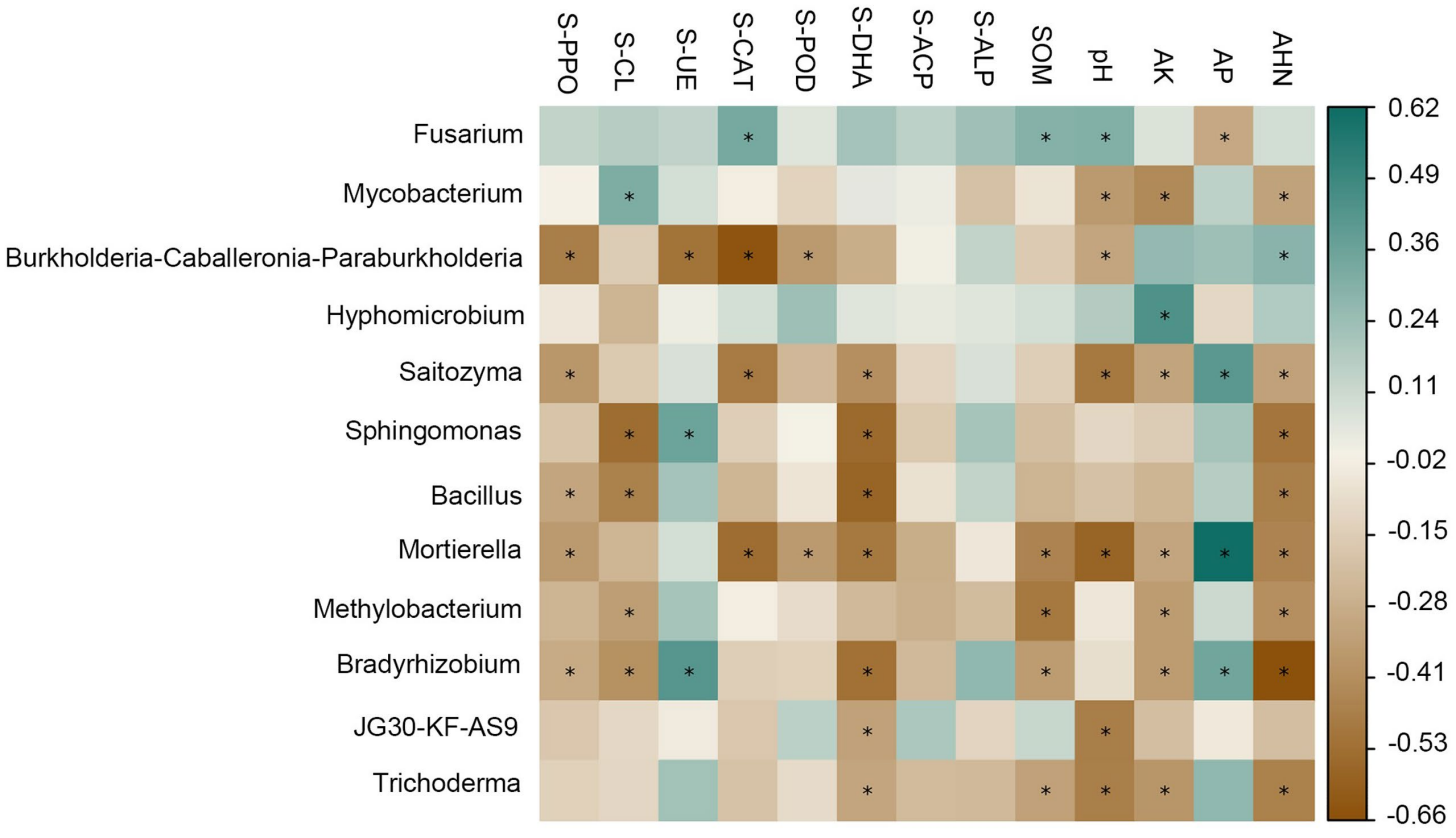
Relationships among soil physic-chemical, enzyme activities and dominant genera relative abundance of bacterial and fungal communities at the genus level. Cyan represents positive correlation, brown represents negative correlation, the darker the color, the stronger the correlation. The symbol “*” indicates statistical significance at *p* < 0.05.

There was a strong correlation between soil physicochemical factors and enzyme activities and the dominant genus of rhizosphere bacteria (Figure 4). For the relative abundance of dominant genera in the bacterial community, there was a significant positive correlation between *Mycobacterium* and S-CL (*R* = 0.32), and a significant negative correlation with pH (*R* = 0.35), AK (*R* = 0.42), and AHN (*R* = 0.31); *Burkholderia-Caballeronia-Paraburkholderia* was negatively correlated with S-PPO (*R* = 0.47), S-CAT (*R* = 0.64), S-POD (*R* = 0.35) and pH (*R* = 0.31), and positively correlated with AHN (*R* = 0.29); *Hypomicrobium* has a significant negative correlation with AK (*R* = 0.45); *Acidothermus* was negatively correlated with S-PPO (*R* = 0.39), S-CAT (*R* = 0.40), S-DHA (*R* = 0.48), pH (*R* = 0.51) and AHN (*R* = 0.32); *Sphingomonas* showed significant negative correlations with S-DHA (*R* = 0.55) and AHN (*R* = 0.50); For the relative abundance of dominant genera in fungal communities, there was a significant positive correlation between *Fusarium* and S-CAT (*R* = 0.34), SOM (*R* = 0.30), pH (*R* = 0.31), and a significant negative correlation with AP (*R* = 0.30). *Mortierella* and S-PPO (*R* = 0.35), S-CAT (*R* = 0.54), S-POD (*R* = 0.35), S-DHA (*R* = 0.49), SOM (*R* = 0.45), pH (*R* = 0.57), AK (*R* = 0.31), AHN (*R* = 0.44) were significantly negatively correlated with each other and positively correlated with AP (*R* = 0.62).

### 3.5. Functional predictions

**Figure 5:**
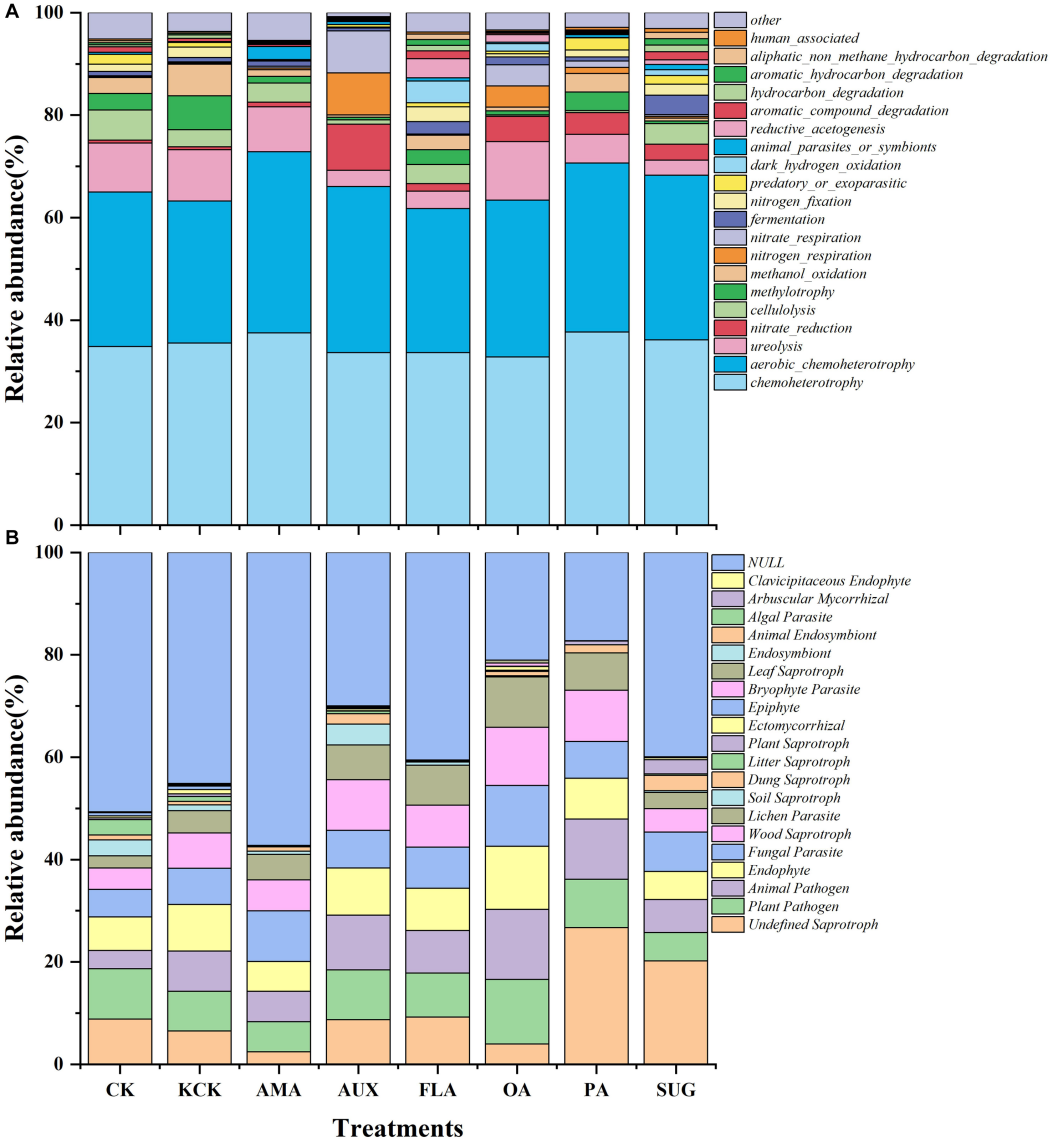
Effects of different root secretions on relative abundance of the top 20 functional genes in soil bacteria **(A)** and fungi **(B)** communities. *n* = 6. CK, control (sterile water); KCK, 20% sterile methanol solution; AMA, amino acids; AUX, auxin; FLA, flavonoids; OA, organic acids; PA, phenolic acids; SUG, sugar.

The functional prediction of the soil bacterial community revealed that the main gene functional groups in each treatment were *chemoheterotrophy* and *aerobic*_*chemoheterotrophy*, which accounted for 66.39% of the total. There were no significant changes in the percentages of these functional groups after the addition of exogenous secretions. In the case of the fungal community, there were no differences between the functional communities of the fungi under the CK and KCK treatments. The *Undefined Saprotroph* also significantly increased by 310.21% in the PA treatment. The addition of various compounds did not significantly alter the relative abundance of the *Plant Pathogen* and *Fungal Parasite* functional groups. However, *Animal Pathogen*, *Endophyte*, *Wood Saprotroph*, and *Lichen Parasite* showed a significant increase in relative abundance in the OA treatment, by 72.90, 35.86, 64.99, and 125.78%, respectively, (all *p* < 0.05; Figure 5; Supplementary Table S4).

**Figure 6:**
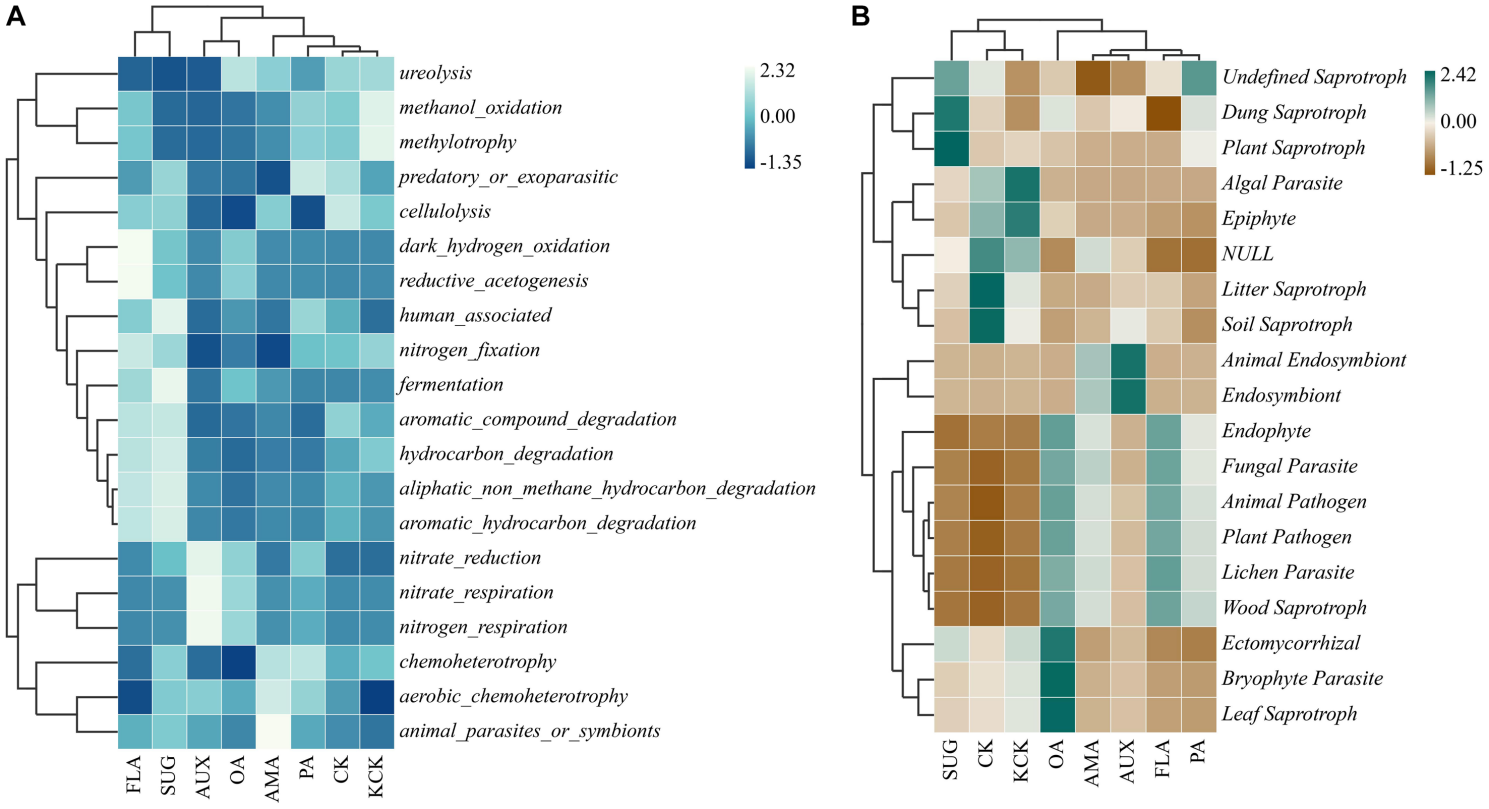
Clustering heat map of the functional diversity of rhizosphere microbial genes, bacterial **(A)** and fungal **(B)**. *n* = 6. CK, control (sterile water); KCK, 20% sterile methanol solution; AMA, amino acids; AUX, auxin; FLA, flavonoids; OA, organic acids; PA, phenolic acids; SUG, sugar.

Based on the functional annotations of the samples in the database and the abundance information, a heat map was created to show the top 20 functions in terms of abundance and their abundance information in each treatment (Figure 6). Among the bacterial functional groups, the cluster analysis revealed that CK, KCK, PA, AMA, AUX, and OA treatments had similar functional community composition, while treatments FLA and SUG were similar but had higher abundance of gene functions such as *automatic*_*compound*_*degradation*, *hydrocarbon*_*degradation*, and *automatic*_*hydrocarbon*_*degradation*. *Nitrate*_*reduction*, *methylotrophy*, and *methanol*_*oxidation* genes had higher relative abundance in other treatments. In the fungal functional groups, the functional community composition of treatments CK, KCK, and SUG were grouped together, while the other treatments were grouped together.

## 4. Discussion

### 4.1. Effects of different root secretions on soil physic-chemical properties and enzyme activities

Soil enzymes play an important role in the degradation and transformation of soil nutrients such as alkali-hydrolyzed nitrogen, and also participate in other physiological and biochemical processes. The activity and diversity of soil enzymes can have a significant impact on the soil physico-chemical properties, such as nutrient cycling, soil structure, and organic matter decomposition (Jing et al., 2020). On one hand, the findings of this study indicate that the addition of compounds significantly increased the activities of S-PPO, S-DHA, S-POD, and S-CAT, suggesting that the root secretions of *Areca catechu* mainly affect redox enzymes (Table 2). Specifically, S-PPO can accelerate the soil humification process, while S-DHA, S-POD, and S-CAT have been found to play crucial roles in soil bio-oxidation (Tan et al., 2017; Telesiński et al., 2019; Li Q. et al., 2022). The four soil enzyme activities mentioned above exhibited a significant positive correlation with soil organic matter (SOM) and pH in this study (Figure 3) supporting the notion that oxidoreductases in lichen can catalyze the formation of alkali-hydrolyzed nitrogen (Beckett et al., 2013). The present study demonstrates that crop root secretions, an important driver of microbial activity and a steady source of soil organic carbon, exert a robust regulatory effect on both soil physicochemical and enzymatic activities (Yan et al., 2023). Through a series of biochemical reactions, various types of secretions are transformed into the nutrients necessary for organisms, providing diverse sources of carbon for the soil microbiome. In the present study, the addition of different root secretions led to significant increases in pH, soil organic matter (SOM), available potassium (AK), and alkali-hydrolyzable nitrogen (AHN), as confirmed by the results presented in Table 1. Interestingly, the noticeable decrease in available phosphorus (AP) observed in this study could be related to the increased activity of catalase. Prior research has indicated that catalase is a key factor that inhibits the conversion of soil organic phosphorus to effective phosphorus (Strickland et al., 2015). Under the absence of exogenous phosphorus interference, the increase in catalase activity led to a significant reduction in soil effective phosphorus content, as demonstrated in this study (Wang et al., 2012). The results of this study suggest that acid phosphatase activity was not affected significantly by the addition of root secretions, which may correspond to a low rate of available phosphorus (AP) accumulation in the soil (Liu et al., 2023).

### 4.2. Effect of different root secretions on the diversity of soil microbial communities

As a crucial mediator of interaction between crop and soil microbial communities, the alterations in both the type and quantity of root secretions could markedly impact the diversity of soil microbial communities (Zhalnina et al., 2018). Root secretions have complex and diverse effects on microbial diversity, potentially simultaneously influencing multiple microbial groups. However, specific compounds in root secretions, such as plant hormones, have been indicated to directly impact microbial growth and metabolism, thereby influencing microbial diversity (Vives-Peris et al., 2020; Zhao et al., 2021). Some flavonoid and organic acid compounds found in root secretions are only mineralized by specific microorganisms, while most soil microorganisms are unable to utilize them, thus affecting soil microbial diversity (Qu and Wang, 2008; Eilers et al., 2010).

The results from Supplementary Table S4 in the Appendix demonstrated that soil microbial diversity was inversely correlated with soil properties, following the application of AUX, OA, and PA. This suggests that the reduction in bacterial and fungal diversity could be attributed to changes in soil properties resulting from these treatments. While the addition of AUX, OA, and PA had a beneficial effect on AK, AHN, and pH in the rhizosphere soil of this study, the ensuing increase in nitrogen and pH levels may have exceeded the tolerable range of certain microbial communities, thereby inducing adverse changes in their living environment that inhibited their growth. Specifically, the pronounced alterations in the soil environment could hinder the growth of microbial populations that had adapted to the original living conditions (Zheng et al., 2022). The rise in soil nitrogen content accelerated the loss of rare species and led to a decline in microbial diversity. While an increase in other nutrients may help certain microorganisms withstand the negative impacts caused by changes in soil nutrient conditions, the overall effect was a decrease in microbial diversity (Wang et al., 2018).

Root secretions exhibit notable spatiotemporal variability during various physiological stages of crops, and the impacts of distinct components on the assembly of microbial structures may differ significantly (Zhalnina et al., 2018). Previous research has demonstrated that the composition of the rhizosphere microbial community is influenced by the composition of root secretions. The significant differences observed in microbial community structure among FLA, PA, and CK in this study were in line with previous findings (Cadot et al., 2021; Figure 1). The selectivity of microorganisms for the consumption of root secretions influences the aggregation of rhizosphere microorganisms. Certain secretions encourage the colonization of specific microorganisms, while others inhibit microbial growth. As such, different types of secretions can have varying impacts on the diversity of rhizosphere microorganisms (Sasse et al., 2018; Siczek et al., 2018; Bao et al., 2022). In this study, FLA and PA may have divergent impacts on the abundance of dominant species, consequently leading to alterations in microbial diversity (Figures 1, 2).

### 4.3. Effect of root secretions on the abundance of dominant microbiome

The reduction in microbial community abundance and diversity observed in response to compounds addition may be attributed to the inhibition of harmful microorganisms and stabilization of microbial structure (Salem et al., 2022). This study found that the addition of compounds leads to alterations in both the diversity and relative abundance of dominant species of soil microbial communities. The interaction between plant root exudates and specific microbial communities is complex and diverse. They can attract or repel microorganisms, modulate symbiotic and antagonistic relationships, thereby influencing the health and growth of plants (Zhang et al., 2022). Specific root secretions can attract beneficial bacteria and reduce the proliferation of pathogenic bacteria, contributing to these changes (Liu A. et al., 2022). Terpenoids and polyketides present in root secretions have been found to suppress phytopathogenic mycobacteria while facilitating the recruitment of specific bacteria to rhizosphere soils (Strickland et al., 2015). Chemical substances in plant root exudates can serve as signaling molecules to attract beneficial microorganisms and establish symbiotic relationships. Among them, phenolic and flavonoid compounds have been shown to enhance the expression of nodulation genes in nitrogen-fixing bacteria (Hirsch et al., 2003).These signaling molecules can trigger chemical reactions in rhizobial bacteria, leading to the formation of root nodules and the establishment of symbiotic relationships with plant roots (Xie et al., 2019).

Therefore, the addition of compounds is more likely to impact bacterial communities rather than fungal communities, as demonstrated in Figure 3 where fungal communities were found to be insensitive to the addition of compounds. Our analysis revealed that the relative abundance of dominant bacterial genera had significant negative correlations with soil physicochemical properties such as S-PPO, S-UE, S-CAT, S-DHA, pH, and AK. This finding suggests that the microbial community in soil that has undergone continuous coffee cropping exhibits sensitivity to changes in soil physicochemical properties and enzyme activities brought about by root secretion addition, which is consistent with the results of previous studies (Liu et al., 2019; Figure 4). The addition of root secretions promotes the interaction between microorganisms, and regulates the structure of soil microbial community by reducing the intensity of competition between microbial communities (Eldridge et al., 2017). In addition, the addition of root secretions also enhances the recruitment of microorganisms compatible with root secretions and reduces the survival space of pre-existing dominant species. This may explain the observed reduction in the abundance of dominant genera (Santoyo, 2022). The results observed in this study demonstrate how the addition of primary exudates from *Areca catechu* roots promotes the transition of soil microbial community structure from imbalance to stability.

### 4.4. Effect of different compounds on functional genes

The structure and function of soil microorganisms are considered to be inseparable, thus the difference of soil microbial communities under compounds addition may change the functional type of these microbial communities (Xie et al., 2022). Soil microbial community function may be influenced by the addition of simulated compounds, and microbial substrate preferences for root metabolites (amino acids and organic acids) can positively aid in the selection of specific functional taxa (Mundra et al., 2022). *Chemoheterotrophy* and *Aerobic chemoheterotrophy* can participate in the carbon cycle and accelerate the decomposition of alkali-hydrolyzed nitrogen (Yu et al., 2021). However, The exogenous secretions did not significantly affect the percentage of *chemoheterotrophic* functional genes in this study (Figure 5).

In this study, the CK and KCK treatments showed a higher proportion of potential pathogenic functional groups such as *Plant Pathogen* and *Fungal Parasite*. These findings suggest that a high relative abundance of pathotrophic fungal functional groups in coffee rhizosphere soils may be the primary cause of CCO (Liu et al., 2019). It is worth noting that the relative abundance of functional groups such as *Plant Pathogen*, *Fungal Parasite*, *Animal Pathogen*, *Endophyte*, W*ood Saprotroph*, and *Lichen Parasite* did not show significant changes after the addition of various compounds (except for organic acid compounds). This indicates that prior to the addition of compounds, the fungal functional groups present in coffee rhizosphere soils were more complex, and the addition of compound compounds led to a decrease in fungal functional group complexity (Figure 5). These results suggest that the potential risk of coffee disease can be mitigated by the addition of secretions from *Areca catechu*.

## 5. Conclusion

This study reveals that adding primary exudates from *Areca catechu* roots can enhance soil fertility and enzyme activity in coffee rhizosphere soils, resulting in significant changes in the structure and function of soil microbial communities. Specifically, the addition of flavonoids, plant hormone, phenolic acids, and sugars can inhibit the growth of bacterial pathogens in continuously cultivated soils by promoting soil nutrient enrichment and increasing soil enzyme activity, thus reducing the risk of coffee diseases. The addition of organic acids and flavonoids may increase the relative abundance of pathogenic genera like *Fusarium* and lead to a significant increase in the relative abundance of pathogenic fungal functional groups. The inclusion of plant hormone, phenolic acids, and sugars could promote the proliferation and relative dynamic balance of non-pathogenic bacteria in continuously cropped coffee rhizosphere soils, which ultimately contributes to overcoming challenges associated with continuous cropping in coffee rhizosphere soils.

## Data availability statement

The data presented in the study are deposited in the Mendeley Data repository, accession number doi: 10.17632/tgfz6h2bxh.1.

## Author contributions

SZ: Conceptualization, Data curation, Formal analysis, Software, Validation, Visualization, Writing – original draft, Writing – review & editing. AZ: Methodology, Writing – review & editing. QZ: Conceptualization, Funding acquisition, Methodology, Project administration, Resources, Supervision, Writing – review & editing. YD: Formal analysis, Methodology, Project administration, Supervision, Writing – review & editing. LS: Formal analysis, Supervision, Writing – review & editing. YS: Formal analysis, Project administration, Supervision, Writing – review & editing. FZ: Supervision, Writing – review & editing. DH: Supervision, Writing – review & editing. WX: Formal analysis, Supervision, Visualization, Writing – review & editing.

## Footnotes

^1^https://docs.qiime2.org/2019.4/tutorials/
