# Supplementary material for: The impact of main Areca Catechu root exudates on soil microbial community structure and function in coffee plantation soils
Source: Front Microbiol. 2023 Oct 20;14:1257164. doi: 10.3389/fmicb.2023.1257164 (PMC10623314; doi:10.3389/fmicb.2023.1257164)
Supplement: Supplementary file 1 [file Table_1.pdf]

Supplementary Material

List of Appendix Captions:

TABLE S1. The composition of various root secretions.

TABLE S2. Relative Abundance of Dominant Microbial Genera in Rhizosphere Soil under Different Treatments.

TABLE S3. Relationship between soil microbial alpha diversity and environmental factors.

TABLE S4. Composition of main functional groups of rhizosphere soil microorganisms under different treatments.

TABLE S1. The composition of various root secretions.

Types of secretion	Component	mg/d	Types of secretion	Component	mg/d
Amino acids	Lysine	2.284	Flavonoids	Apigenin	1.689
	Serine	3.284		Hesperidin	1.783
	Valine	2.196		Coumarin	1.522
	Cysteine	3.787		Naringin	1.702
plant hormone	Indole acetic acid	2.189	Sugars	Glucose	3.753
	Methyl indole acetate	2.150		Sucrose	3.565
	Gibberellin	2.278		Arabinose	3.753
Organic acids	Citric acid	4.002	Phenolic acids	P-hydroxybenzoic acid	2.467
	Tartaric acid	4.690		Salicylic acid	2.466
	Succinic acid	3.690		Ferulic acid	2.427

TABLE S2. Relative Abundance of Dominant Microbial Genera in Rhizosphere Soil under Different Treatments.

Genus(%)	CK	KCK	AMA	AUX	FLA	OA	PA	SUG
Bacterial								
Myo	32.82±14.17^{a}	29.8±14.32a	43.42±27.28a	7.9±10.95b	5.88±3.48b	43.55±26.98a	1.34±1.32b	4.77±2.97b
BCP	3.49±2.24c	1.81±1.51c	3.76±3.81c	53.15±25.55a	26.04±20.56b	0.2±0.18c	1.45±1.2c	11.83±23.44bc
Hyp	1.54±1.39c	11.46±9.95abc	6.62±7.66c	6.73±2.84c	9.72±7.8bc	2.5±1.47c	24.3±14.06a	22.47±18.33ab
Aci	12.21±11.26a	7.7±3.17ab	7.55±8.14ab	1.01±0.81b	5.73±3.5ab	0.41±0.27b	0.91±0.53b	7.75±8.24ab
Sph	3.01±1.43b	3.34±1b	0.48±0.5b	0.88±0.64b	3.05±2.83b	0.84±0.7b	3.08±2.78b	7.88±3.34a
Bac	4.55±3.03a	2.96±1.39abc	1.03±0.46c	0.98±0.65c	3.14±1.8ab	0.92±0.77c	2.38±1.09bc	3.21±0.96ab
Met	3.13±2.94b	3.62±2.64b	0.01±0.01b	0.43±0.88b	0.21±0.14b	0.27±0.36b	9.64±6.4a	0.25±0.33b
Bra	3.31±1.91a	2.72±1.6ab	0.09±0.14c	0.18±0.13c	2.32±1.91ab	0.94±0.62bc	1.71±1.52abc	2.46±1.7ab
JKA	2.02±1.09ab	1.68±0.7ab	4.05±4.53a	0.11±0.08b	1.35±1.01b	0.15±0.11b	0.32±0.22b	2.3±1.17ab
Fungi								
Fus	11±7.08c	18.22±6.23bc	40.91±30.25^{abc}	27.18±31.5bc	66.45±21.11a	61.97±35.88a	47.58±33.42ab	20.67±13.03bc
Sai	20.96±14.14a	10.39±4.75ab	11.71±17.94ab	4.15±3.27b	11.4±7.31ab	1.71±1.79b	0.05±0.02b	7.42±7.3b
Mor	10.34±6.07a	3.32±1.77b	0.24±0.25b	1.71±2.02b	1±2.06b	0.24±0.32b	0.02±0.01b	1.49±1.66b
Pen	1.86±2.34a	1±1.01a	2.6±4.5a	4.69±7.61a	0.69±0.85a	0.61±0.61a	2.49±2.48a	0.81±0.81a
Tri	1.38±1.01b	3.27±1.56a	0.29±0.17bc	0.28±0.45bc	0.09±0.11c	0.38±0.24bc	0.26±0.54bc	1.21±1.28bc

NOTE:*Myo:Mycobacterium,BCP:Burkholderia-Caballeronia-Paraburkholderia,Hyp:Hyphomicrobium,Aci:Acidothermus,Sph:Sphingomonas,Bac:Bacillus,Met:Methylobacterium,Bra:Bradyrhizobium,JKA:JG30-KF-AS9,Fus:Fusarium,Sai:Saitozyma,Mor:Mortierella,Pen:Penicillium,Tri:Trichoderma*. Different letters indicate significant differences between treatments under the same soil microbes ($p < 0.05$).

TABLE S3. Relationship between soil microbial alpha diversity and environmental factors.

Indicators	Bacteria Shannon	Fungi Shannon
S-PPO	-0.38*	-0.29*
S-CL	-0.25	-0.21
S-UE	0.14	0.22
S-CAT	-0.36*	-0.37*
S-POD	-0.19	-0.31
S-DHA	-0.5*	-0.39*
S-ACP	-0.09	-0.25
S-ALP	-0.04	-0.08
SOM	-0.31*	-0.42*
pH	-0.52*	-0.47*
AK	-0.4*	-0.31*
AP	0.4*	0.47*
AHN	-0.53*	-0.52*

* Correlation is significant at the 0.05 level; n=6， treatments abbreviations and indicator abbreviations see table 2 and table 3.

TABLE S4. Composition of main functional groups of rhizosphere soil microorganisms under different treatments.

Gene functional (%)	CK	KCK	AMA	AUX	FLA	OA	PA	SUG
Bacterial								
chemoheterotrophy	34.82±2.24[a]	35.49±0.81[a]	37.49±3.07[a]	33.61±6.04[a]	33.66±6.4[a]	32.8±5.4[a]	37.68±4.94[a]	36.13±5.88[a]
aerobic_chemoheterotrophy	30.16±5.22[a]	27.71±3.03[a]	35.33±3.42[a]	32.44±5.69[a]	28.07±6.65[a]	30.6±6.44[a]	32.94±5.49[a]	32.15±9.99[a]
Fungi								
NULL	50.68±18.47[ab]	45.17±14.1[abc]	57.24±16.24[a]	29.98±17.01[bcd]	40.54±7.7[abcd]	21.07±20.25[cd]	17.29±13.87[d]	39.93±27.61[abcd]
Undefined Saprotroph	8.79±3.75[bc]	6.5±1.76b[c]	2.4±1.92[c]	8.7±7.53[bc]	9.21±10.77[bc]	3.93±3.32[c]	26.68±18.98[a]	20.16±17.56[ab]
Plant Pathogen	9.89±12.73[a]	7.71±2.38[a]	5.87±3.31[a]	9.75±2.62[a]	8.59±2.37[a]	12.63±3.83[a]	9.46±7.59[a]	5.53±2.57[a]
Animal Pathogen	3.52±1.81[d]	7.9±2.4[bcd]	5.96±3.28[cd]	10.67±2.95[abc]	8.31±2.41[bcd]	13.66±5.18[a]	11.74±5.86[ab]	6.49±3.83[bcd]
Endophyte	6.57±2.03[b]	9.1±2.32[ab]	5.86±3.28[b]	9.23±2.62[ab]	8.29±2.67[ab]	12.37±4.02[a]	7.98±5.36[ab]	5.46±2.62[b]
Fungal Parasite	5.4±3.55[a]	7.07±2.54[a]	9.86±9.92[a]	7.32±3.83[a]	7.98±2.57[a]	11.86±5.29[a]	7.21±4.84[a]	7.69±5.27[a]
Wood Saprotroph	4.18±2.32[c]	6.88±2.41[abc]	6.06±2.32[abc]	9.9±4.04[ab]	8.21±2.46[abc]	11.35±4.42[a]	9.99±7.3[ab]	4.62±2.03[bc]
Lichen Parasite	2.34±1.26[c]	4.36±2.42[bc]	4.99±3.05[abc]	6.79±4.15[abc]	7.85±2.58[ab]	9.83±5.72[a]	7.3±4.93[abc]	3.15±1.94[bc]

NOTE: Different letters indicate significant differences between treatments under rhizosphere soil main functional groups ($p < 0.05$).
